# Rotator Cuff Tears: Correlation Between Clinical Examination, Magnetic Resonance Imaging and Arthroscopy

**DOI:** 10.7759/cureus.56065

**Published:** 2024-03-12

**Authors:** Chiranjeevi S Gowda, Kiyana Mirza, Dev A Galagali

**Affiliations:** 1 Trauma and Orthopaedics, The Rotherham NHS Foundation Trust, Rotherham, GBR; 2 Trauma and Orthopaedics, Norfolk and Norwich University Hospitals NHS Foundation Trust, Norwich, GBR; 3 Foot and Ankle Surgery, Indian Foot and Ankle Society, Bangalore, IND

**Keywords:** shoulder pain, rotator cuff, physical examination, magnetic resonance imaging, arthroscopy

## Abstract

Background

Arthroscopy in diagnosing a rotator cuff injury has surgical and anaesthesia-related risks. Magnetic resonance imaging (MRI), a non-invasive procedure, is expensive, and lacks dynamic components making it less favourable. Clinical examination narrows the diagnosis, but lacks diagnostic accuracy due to overlap of clinical signs and symptoms. We aimed to determine the diagnostic accuracy of clinical examination and MRI in rotator cuff tears by correlating it with arthroscopy.

Methods

This prospective, cross-sectional validation study included patients (N=28) with shoulder pain with clinical characteristics suggestive of rotator cuff tears. Clinical diagnoses and MRI were done preoperatively, following which each patient underwent arthroscopic surgery. Shoulder arthroscopy findings were correlated with those of clinical examination and MRI.

Results

The mean age of patients was 50.21±9.66 years, with 60.71% being males. Clinical examination was 100% sensitive and 73.8% specific for detecting rotator cuff tears. MRI was 92.85% sensitive and 98.8% specific in detecting rotator cuff tears. Shoulder MRI demonstrated a higher agreement with arthroscopy than clinical results for subscapularis, supraspinatus, infraspinatus, teres, and biceps tendon appearance.

Conclusion

MRI results in identifying rotator cuff pathologies are comparable with arthroscopy. Clinical examination findings are variable due to an examiner’s bias and therefore its diagnostic scope is limited. However, clinical examination with MRI together might accurately identify the rotator cuff injury.

## Introduction

The shoulder is a complex joint, anatomically, functionally and biomechanically. It consists of four joints - glenohumeral, acromioclavicular, scapulothoracic, and sternoclavicular joints. Glenohumeral joint is the most mobile joint in the body with six degrees of freedom. This leads to increased predisposition to dislocations and the need for multiple stabilizers. These include static and dynamic stabilizers. Static stabilizers are the capsuloligamentous structures, glenoid and the labrum. Dynamic stabilizers are the conjoint tendon, long head of the biceps and most importantly the rotator cuff muscles [1].

Rotator cuff muscles comprising of the supraspinatus, infraspinatus, teres minor and subscapularis, play a key role in the concavity-compression concept of stability. They stabilize the shoulder anteroposteriorly [1]. Rotator cuff muscles enable movements of the glenohumeral joint. Supraspinatus depresses the scapula and acts in synchrony with the deltoid muscle to abduct the arm at the shoulder. Infraspinatus aids in the external rotation of the shoulder along with teres minor, provides restraint against the anterior dislocation of the humerus during abduction, and medially rotates the humerus. Due to the major contribution of the rotator cuff group of muscles to glenohumeral joint motion, any damage or tear of these structures will result in a drastic decline of shoulder function.

Rotator cuff tears occur in 9.2% of patients less than 20 years of age and range to more than 62% in patients more than 80 years of age [2]. These patients may present with a history of trauma after which they have difficulty in moving the shoulder or a vague history of insidious onset occasional shoulder pain with difficulty in overhead activity. Clinical examination is the basis upon which an orthopaedic surgeon is able to arrive at a preliminary diagnosis of a rotator cuff pathology in the outpatient setting. Due to the vague symptomatology and overlap of clinical findings among various shoulder pathologies, the accuracy of clinical examination of the shoulder has been brought into question [3-5]. Various imaging modalities like magnetic resonance imaging (MRI), magnetic resonance arthrography (MRA) and ultrasonography (USG) have been used for radiological diagnosis of rotator cuff tears. MRI is the preferred test which can assess tendinopathy, and partial and complete tears [4,6]. While MRI is a non-invasive investigation with relatively few contraindications, its high cost and lack of dynamic visualization of the joint make it a less feasible investigation in some clinical settings for the diagnosis of a rotator cuff pathology. Arthroscopic examination is, however, the gold standard diagnostic tool for rotator cuff pathologies [7]. While its accuracy is undisputed, its invasive nature and anaesthesia-related risks make it not only expensive, but less practical in many situations. A multitude of patients visit OPD regularly with shoulder pain making it crucial to devise and formulate a practical, cost-effective and efficient method to arrive at a diagnosis for rotator cuff pathologies. Therefore, the aim of this study is to determine the diagnostic accuracy of clinical examination and MRI in rotator cuff tears by correlating it with arthroscopy.

## Materials and methods

This prospective, cross-sectional validation study was done at a tertiary care hospital between November 2018 and April 2020. It was conducted among 28 consecutive adults with clinical or radiological characteristics suggestive of rotator cuff tears, after clearance from the institutional ethical committee and informed consent of the patients. Patients with instability disorders, contraindication to MRI, previous history of shoulder surgery, fractures in the shoulder, and adhesive capsulitis were excluded.

**Figure 1:**
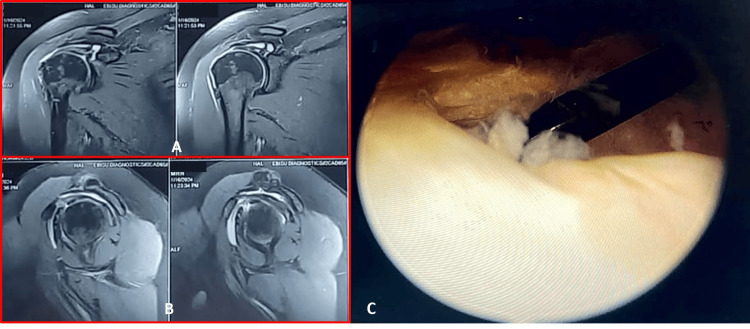
MRI and arthroscopy findings of a rotator cuff A - Coronal section MRI showing a rotator cuff tear B - Sagittal section MRI showing a supraspinatus tear C - Arthroscopic examination showing a supraspinatus tear

All patients underwent clinical examination which included five key tests, one for each of the muscles and one for the biceps tendon by a single assessor, a consultant orthopaedic surgeon. Five tests are the lift-off test (subscapularis), Jobe’s test (supraspinatus), Hornblower’s test (teres minor), External rotation lag sign at 90 degrees (infraspinatus) and Speed’s test (long head of biceps tendon). These tests were performed as per standard protocols described by Jain et al. [8]. Both the patient and the assessor were blinded with regard to the diagnosis. MRI was performed preoperatively using a 1.5 T scanner (PHILIPS ACHIEVA) with a dedicated 16-channel system coils studied in different sequences - T1W, T2W, T2 STIR and PDW. All MRIs were assessed by a single radiologist who was blinded with regard to the clinical findings. Patients then underwent arthroscopic examination and required interventions by a single arthroscopy surgeon. The results of clinical examination, MRI and arthroscopic diagnosis were tabulated and correlated (Figure 1).

Data was analysed using R v4.1.2. Categorical variables were given in the form of frequency and continuous variables were given in Mean ± SD/ Median (Min, Max). Diagnostic parameters (Sensitivity, Specificity, negative predictive value [NPV], and positive predictive values [PPV]) are calculated for clinical and MRI results with the arthroscopy results. Kappa agreement was checked for clinical and MRI results with the arthroscopy results to evaluate the interrater reliability for dichotomous variables. Kappa statistics expresses the degree of agreement between two raters corrected for chance agreement. Value ≤0.4 was considered as fair to poor, 0.41-0.60 as moderate, 0.61-0.80 as substantial and >0.80 as excellent/almost perfect, P≤0.05 indicates statistical significance.

## Results

Subjects’ age ranged from 35-68 years with a mean age of 50.21 ± 9.66 years. Out of 28 subjects, 17 (60.71%) were males and 11 (39.29%) were females with a gender ratio of 1.55:1.

Supraspinatus tear

MRI showed a sensitivity of 100% in diagnosing supraspinatus tendon tear with 100% PPV and NPV, while specificity (100%) and NPV (96.43%) were seen with clinical diagnosis. Shoulder MRI significantly showed a better agreement with arthroscopy than a clinical diagnosis for supraspinatus tendon appearance, which showed insignificant agreement (k = 1 vs. k= 0).

Infraspinatus tear

MRI showed a sensitivity of 100%, specificity of 80%, PPV of 90%, and NPV of 100% in diagnosing infraspinatus tendon tear. Specificity was 100% and PPV was 100% with a clinical diagnosis but MRI correlated better with arthroscopy than a clinical diagnosis for infraspinatus tendon appearance (k = 0.8372 vs. k= 0.7143).

Teres minor tear

MRI had sensitivity of 100% and PPV of 96.43% in diagnosing teres tendon tear. However, both shoulder MRI and clinical diagnosis showed poor agreement with arthroscopy for teres minor appearance (k = 0 vs. k= 0.2391).

Subscapularis tear

MRI is more sensitive (92.31% vs. 61.54%) in diagnosing subscapularis tendon with NPV of 93.33% vs. 75% for clinical diagnosis. Shoulder MRI showed a significant agreement with arthroscopy than a clinical diagnosis for subscapularis tendon appearance, which showed substantial agreement (k = 0.8564 vs. k = 0.6316).

Biceps tendon tear

MRI showed a sensitivity of 100%, specificity of 100%, PPV of 100%, and NPV of 100% in diagnosing biceps tendon tear. Also, shoulder MRI showed better agreement with arthroscopy than a clinical diagnosis for biceps tendon appearance, which showed an insignificant poor correlation (k = 1 vs. k = 0.3553).

Cumulative statistics demonstrated clinical examination was 100% sensitive and 73.8% specific in detecting rotator cuff tears with a PPV of 71.79% and NPV of 100%. MRI was 92.85% sensitive and 98.8% specific in detecting rotator cuff tears, with a PPV of 98.11% and NPV of 95.4% (Table 1).

**Table 1:** Sensitivity and specificity of MRI and clinical findings of each part of rotator cuff when compared to arthroscopy findings Categorical variables were given in the form of frequency and continuous variables were given in Mean ± SD/ Median (Min, Max). Diagnostic parameters (Sensitivity, Specificity, negative predictive value [NPV], and positive predictive values [PPV]) are calculated for clinical and MRI results with the arthroscopy results. Kappa agreement was checked for clinical and MRI results with the arthroscopy results to evaluate the interrater reliability for dichotomous variables.

Variables	Arthroscopy				Sensitivity (95% CI)		Specificity (95% CI)			PPV (95% CI)		NPV (95% CI)		Kappa (p-value)
	Intact		Tear											
Biceps clinical														
Intact	18 (72%)		0		72% (50.61% - 87.93%)		100% (29.24% - 100%)			100% (81.47% - 100%)		30% (6.67% - 65.25%)		0.3553 (0.0673)
Tear	7 (28%)		3 (100%)											
Biceps MRI														
Intact	25 (100%)		0		100% (86.28% - 100%)		100% (29.24% - 100%)			100% (86.28% - 100%)		100% (29.24% - 100%)		1 (0.005*)
Tear	0		3 (100%)											
Infraspinatus clinical														
Intact	14 (77.78%)		0		77.78% (52.36% - 93.59%)		100% (69.15% - 100%)		100% (76.84% - 100%)			71.43% (41.9% - 91.61%)		0.7143 (<0.001*)
Tear	4 (22.22%)		10 (100%)											
Infraspinatus MRI														
Intact	18 (100%)		2 (20%)		100% (81.47% - 100%)		80% (44.39% - 97.48%)		90% (68.30% - 98.77%)			100% (63.06% - 100%)		0.8372 (<0.001*)
Tear	0		8 (80%)											
Teres clinical														
Intact	22 (81.48%)		0		81.48% (61.92% - 93.7%)		100% (2.5% - 100%)		100% (84.56% - 100%)			16.67% (0.42% - 64.12%)		0.2391 (0.2417)
Tear	5 (18.52%)		1 (100%)											
Teres MRI														
Intact	27 (100%)		1 (100%)		100% (87.23% - 100%)		0% (0% - 97.5%)		96.43% (81.65% - 99.91%)			-		0 (0.5)
Tear	0		0											
Supraspinatus clinical														
Intact	0		0		0% (0% - 97.5%)		100% (87.23% - 100%)		-			96.43% (81.65% - 99.91%)		0 (0.5)
Tear	1 (100%)		27 (100%)											
Supraspinatus MRI														
Intact	1 (100%)		0		100% (2.5% - 100%)		100% (87.23% - 100%)		100% (2.5% - 100%)			100% (87.23% - 100%)		1 (0.0075*)
Tear	0		27 (100%)											
Subscapularis clinical														
Negative		8 (61.54%)		0		61.54% (31.58% - 86.14%)		100% (78.2% - 100%)			100% (63.06% - 100%)	75% (50.9% - 91.34%)	0.6316 (< 0.001*)	
Positive		5 (38.46%)		15 (100%)										
Subscapularis MRI														
Intact		12 (92.31%)		1 (6.67%)		92.31% (63.97% - 99.81%)		93.33% (68.05% - 99.83%)			92.31% (63.97% - 99.81%)	93.33% (68.05% - 99.83%)	0.8564 (< 0.001*)	
Tear		1 (7.69%)		14 (93.33%)										

## Discussion

A multidisciplinary approach encompassing constant update of knowledge, understanding and healthy feedback between the surgeon and the radiologist is a step towards accurate diagnosis. MRI has high sensitivity and specificity for diagnosing rotator cuff tear, but tears have been found even in asymptomatic individuals [9-11]. Thus, imaging findings alone are deficient to diagnose a rotator cuff tear and clinic-radiological correlation is an important component in the diagnosis of this clinical syndrome [12]. Hence, in this study, the comparison of imaging and clinical criteria with a gold standard of direct visualization with arthroscopic surgery for the diagnosis of rotator cuff tear was performed. When considering agreement, sensitivity, specificity, PPV and NPV, this data suggests that MRI is better at predicting supraspinatus, infraspinatus, sub-scapularis, teres, and biceps tendon appearance than clinical diagnosis.

It was known that clinical tests had a limited role in diagnosing rotator cuff injuries with poor correlation between findings of clinical examination and arthroscopy [13]. Due to the vast lack of uniformity in findings, extreme variety in the performance, and interpretation of clinical tests for shoulder diseases, are posing a great barrier for the synthesis of data and/or its therapeutic relevance [14]. In this study, clinical examination had a sensitivity of 100%, specificity of 73.8%, PPV of 71.79% and NPV of 100% in diagnosing rotator cuff pathologies. Furthermore, we found clinical tests utilized in the assessment of rotator cuff injuries were least specific for supraspinatus and subscapularis. Consistent with these findings, Jain et al. demonstrated that the lift-off test had a sensitivity and specificity of 17-100% and 60-98% respectively and Jobe’s test had high sensitivity (53-89%) [8]. External rotation lag test for testing the infraspinatus also had similar results to this study with a sensitivity and specificity of 49-98% and 72-98% respectively, against these findings of sensitivity and specificity of 100% and 77%, respectively. Comparable to these results, Hornblower’s sign for teres minor had a sensitivity and specificity of 100% and 93%, respectively. Lastly, the speed test for biceps brachii had a sensitivity and specificity of 53% and 67% respectively while in this study had fairer results with 100% sensitivity and 73% specificity, respectively [8]. Östör et al. reported specificity of lift-off and speed tests were 67% and 75% respectively in compliance with the present study which showed a specificity of 61% and 73%, respectively. Jobe’s test was found to be highly sensitive (94%) and it was comparable with this study's results (100% sensitivity) [15]. In Parra et al. study, clinical examination for rotator cuff tears had a sensitivity and specificity of 68.4% and 100% respectively with PPV of 92.3% [16]. Jain et al. concluded that Jobe’s test and full can test had high sensitivity and specificity for supraspinatus tears and Hornblower’s sign performed well for infraspinatus tears and in general, special tests described for subscapularis tears have high specificity but low sensitivity [17]. The wide range of specificity and sensitivity could be attributed to differences in the patient group, sample size, and reference standards employed in the studies.

The sensitivity and specificity of MRI in this study in detecting rotator cuff tears was like other studies, but MRI was found to be comparatively accurate as compared to other previous studies [10,15,18-20]. MRI was most sensitive in diagnosing supraspinatus and biceps brachii pathologies and least sensitive in diagnosing teres minor tears. MRI was highly sensitive in diagnosing tears of all the muscles of the rotator cuff with subscapularis having the lowest specificity of 92.3%. Ostör et al. showed that the sensitivity of MRI in detecting a pathology of the supraspinatus and infraspinatus tendons were 90.9% and 72% respectively, much like this study where we found a sensitivity of 100% and 80%, respectively [15]. Momenzadeh et al. concluded that MRI was highly sensitive (90.9%) and specific (91.7%) for supraspinatus much like this study (sensitivity and specificity of 100%) [18]. The specificity of MRI for subscapularis, infraspinatus and biceps was found to be 91.3%, 98.5% and 96.9% respectively comparable to this study with specificity of 92%, 100% and 100%, respectively [18]. Sharma et al. revealed MRI was highly sensitive and specific with 93.1% of accuracy for full and 91.1% for partial thickness tears in diagnosing rotator cuff tears [10]. Liu et al. found that high-field MRI had the best diagnostic value with sensitivity of 84% and specificity of 86%, comparable to the high sensitivity and specificity of MRI over clinical examination as noted in this study as well [20]. The accuracy of diagnosis may also be increased by the usage of higher magnetic strength like 3T MRI and MR arthrography [21].

Thus, this study shows that clinical and radiological evaluation of shoulder pathologies go hand-in-hand and not independently prior to diagnosis of rotator cuff pathologies. Hence, appropriate clinical assessment and delivering comprehensive clinical findings to radiologists might increase the accuracy of MRI to diagnose shoulder injuries. However, most clinical tests used are deemed to be positive on eliciting a painful response from the subject, which can be caused by any shoulder pathology. This is a hindrance to arriving at an accurate and reliable clinical diagnosis. This situation is compounded further by the possibility of patients having multiple co-existing lesions that cannot be defined clinically. It therefore is evident that combinations of tests along with MRI will accurately predict the presence of a rotator cuff tear.

The present study is limited in that there may be a selection bias while choosing subjects, as all patients included had rotator cuff injuries severe enough to warrant arthroscopic surgery. We also failed to include individuals who were unable to undergo MRI because of various contraindications like claustrophobia, metallic implants, or pacemaker implants as well as those patients who could not undergo arthroscopy in view of their pre-existing comorbidities. Another limitation is that pain during clinical examination may arise from various pain generators in the shoulder joint which may give rise to a false positive test.

## Conclusions

Rotator cuff tears, beginning to be increasingly common due to a spurt in sports activities around the world, need to be identified, diagnosed and treated. Clinical examination and MRI play a major role in this regard. Our study shows that MRI is a good investigation of choice and results of the same are comparable to arthroscopy which is the gold standard. While clinical examination has better sensitivity, it undeniably lacks specificity as this varies based on the expertise and experience of the examining surgeon. As many shoulder pathologies mimic each other in terms of clinical presentation, it is shown that the number of false positives is high. Hence, a thorough and comprehensive workup of the patient integrating clinical examination along with the use of MRI will aid in the accurate diagnosis of rotator cuff pathologies.
